# Autologous osteochondral plug transplantation for osteochondrosis of the second metatarsal head: a case report

**DOI:** 10.1186/1752-1947-5-308

**Published:** 2011-07-13

**Authors:** Issei Nagura, Hiroyuki Fujioka, Takeshi Kokubu, Masahiro Kurosaka

**Affiliations:** 1 Department of Orthopaedic Surgery, Kobe University Graduate School of Medicine, 7-5-1, Kusunoki-cho, Chuo-ku, Kobe, 650-0017 Japan; 2 Department of Orthopaedic Surgery, Hyogo College of Medicine, 1-1, Mukogawa-cho, Nishinomiya, Hyogo, 663-8501 Japan

## Abstract

**Introduction:**

Osteochondrosis of the second or third metatarsal head is a rare condition called Freiberg's disease. To relieve foot pain, conservative treatment with a foot orthosis to reduce weight-bearing and immobilize the foot are recommended. In cases in which such treatments have proved to be ineffective, several surgical treatments have been performed. The appropriate surgical treatment for Freiberg's disease remains controversial.

**Case presentation:**

We describe the case of a 20-year-old Japanese woman with a three-year history of right forefoot pain and no history of trauma. Two years after treatment by autologous osteochondral plug transplantation, she has neither complaints nor symptoms.

**Conclusion:**

Autologous osteochondral plug transplantation represents a potentially successful surgical arthroplastic option in preserving the metatarsophalangeal joint in patients with Freiberg's disease.

## Introduction

Osteochondrosis of the second or third metatarsal head is a rare condition called Freiberg's disease [1-3]. Generally, to relieve the associated foot pain, conservative treatment including a foot orthosis to reduce weight-bearing and immobilize the foot are recommended. These are especially effective in the early stages of Freiberg's disease [3]. However, in cases where these treatments have resulted in failure, several surgical treatments, such as synovectomy, osteotomy, and excision, have been performed. There is still some controversy concerning the appropriate surgical treatment for Freiberg's disease [3]. We report a case of Freiberg's disease treated by autologous osteochondral plug transplantation.

## Case report

**Figure 1:**
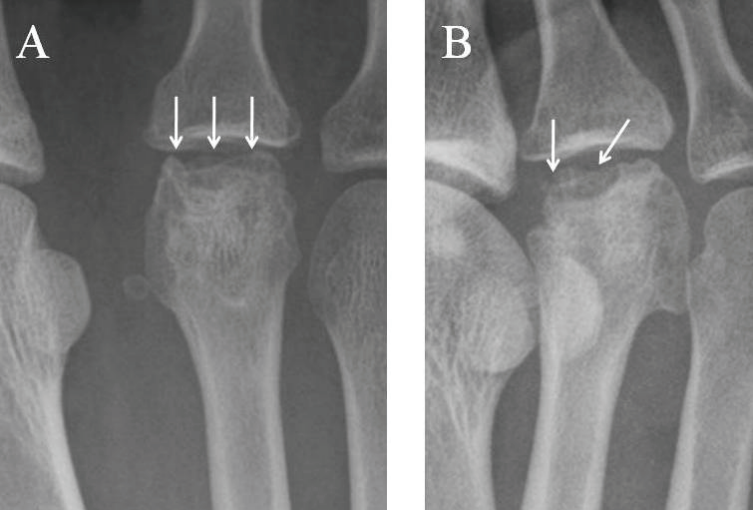
**Radiograph of the patient's metatarsophalangeal (MTP) joint of the right second toe**. **(A) **Anteroposterior and **(B) **oblique views of the MTP joint show that the second metatarsal head was flattened and sclerotic and also depict the irregularity of the joint surface of the head.

**Figure 2:**
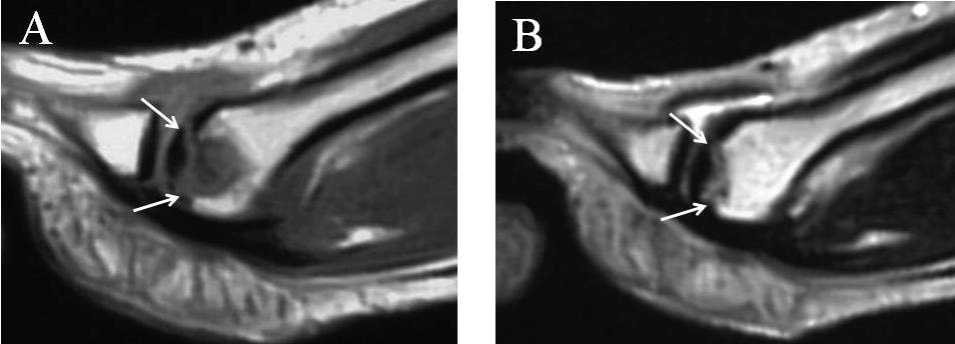
**Magnetic resonance imaging (MRI) studies of the metatarsophalangeal joint of the right second toe**. **(A) **A low-intensity zone was detected within the second metatarsal head in the sagittal plane of the T1-weighted MRI scan. **(B) **A high-intensity zone was detected at the subchondral bone in the sagittal plane of the T2-weighted MRI scan.

We report the case of a 20-year-old Japanese woman who had experienced right forefoot pain for three years while walking and had no history of trauma or any predisposing factors. Her physical examination revealed a slight diffuse swelling on the anterior dorsal region of the foot and tenderness at the second metatarsal head. Dorsal and plantar flexion of the second metatarsophalangeal (MTP) joint were 10 and 30, respectively. The patient admitted a limitation of range of motion and pain on dorsal flexion. A radiograph showed that the second metatarsal head was flattened and sclerotic (Figure 1). Magnetic resonance imaging (MRI) showed a low-intensity zone within the second metatarsal head in the sagittal plane of the T1-weighted image (Figure 2A) and a high-intensity zone at the subchondral bone in the sagittal plane of the T2-weighted image (Figure 2B). These findings suggested that an osteochondral fragment was detached from the subchondral bone in association with osteonecrosis of the second metatarsal head; however, the articular cartilage surface of the second proximal phalanx remained smooth. We diagnosed the patient with Freiberg's disease stage III on the basis of Smillie's classification [2,4]. She had been treated conservatively with a metatarsal dome for six months, but this treatment had not alleviated the pain. Therefore, we treated her surgically by autologous osteochondral plug transplantation.

Through a dorsal skin incision over the second MTP joint, the extensor tendon was retracted and the joint was opened. The articular cartilage of the second metatarsal head was found to be detached from the subchondral bone. The damaged articular cartilage was removed, and a cylindrical osteochondral hole was made in the subchondral bone using the Osteochondral Autograft Transfer System (Arthrex, Naples, FL, USA). An osteochondral graft (6 mm in diameter and 14 mm in depth) was harvested from the femoral condyle and transplanted firmly into the deep defect.

**Figure 3:**
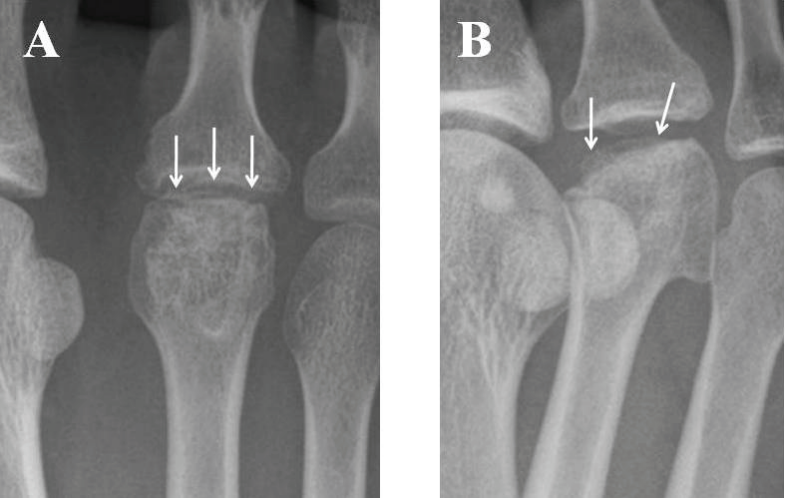
**Radiographic images of the patient's metatarsophalangeal (MTP) joint two years after her operation**. **(A) **Anteroposterior and **(B) **oblique views of the MTP joint of the right second toe show that the transplanted osteochondral graft was united well with the host bone of the second metatarsal head and that the articular surface of the graft had remodeled well.

**Figure 4:**
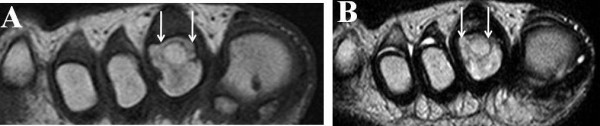
**Magnetic resonance imaging (MRI) study of the metatarsophalangeal joint one year after the operation**. **(A) **No low-intensity zone was detected in the second metatarsal head in the axial plane of the T1-weighted MRI scan. **(B) **No high-intensity zone was detected at the subchondral zone in the axial plane of the T2-weighted MRI study.

After surgery, a short leg cast was applied for four weeks and partial weight-bearing was allowed. Two years after surgery radiography showed that the transplanted graft was well united with the second metatarsal head, that the articular surface of the graft had been remodeled (Figure 3), and that the patient was asymptomatic. MRI showed that no low-intensity zone was detected in the second metatarsal head in the axial plane of the T1-weighted image (Figure 4A), and no high-intensity zone was detected at the subchondral zone in the axial plane of the T2-weighted image (Figure 4B). The dorsal and plantar flexion of the second MTP joint were 30 each, and the patient's American Orthopaedic Foot and Ankle Society (AOFAS) score had improved from 64 to 95 two years after her operation.

## Discussion

Freiberg's disease is a rare osteochondrosis of the second or third metatarsal head, and its etiology is not well understood. Lack of arterial supply and mechanical stress to the metatarsal head is speculated to be the probable etiology [1-3]. The classification of Freiberg's disease is differentiated from stage I (fissure fracture) to stage V, with the latter being the final stage of flattening deformity of the metatarsal head and osteoarthritis of the MTP joint [2-4].

Excision of the proximal phalangeal base and/or the metatarsal head, which is technically easy to perform, has a possibility of risk of progressive hallux valgus, metatarsal pain, and shortening of the toe [3]. Prosthetic joint replacement is a final option for late-stage Freiberg's disease, but possible implant failure and the risk of synovitis risk exist because the typical patient with Freiberg's disease is an adolescent girl with a high physical activity level [3]. An acceptable result was achieved in our patient by performing dorsiflexion osteotomy of the metatarsal bone, the surgical concept of which is that the dorsal necrotic region is rotated and replaced by the intact plantar segment of the metatarsal head [3-5]. This is a reliable treatment option for Freiberg's disease; however, it carries the risk of necrotic changes in the metatarsal head. In addition, remodeling using this technique cannot re-create an anatomically normal articular surface. Recently, autologous osteochondral plug transplantation has been developed, which has had good results for the treatment of cartilaginous lesions of the femoral condyle of the knee and reconstruction of the articular surface to its normal shape without any internal fixation of the defect [6]. Miyamoto *et al. *[7] described four cases of Freiberg's disease, stage III or IV, treated with osteochondral transplantation. An autologous osteochondral plug harvested from the femoral condyle was transplanted into the hole made in the damaged second metatarsal head, and satisfactory results were obtained in all cases. In the present report, we describe treatment of a patient who had stage III Freiberg's disease with autologous osteochondral plug transplantation. We used a relatively large, cylindrical osteochondral graft (6 mm in diameter) harvested from the femoral condyle, because it is important that the plug should be as large as possible to ensure a press-fit into the defect. In the present case, the patient's foot pain was alleviated, the graft was united with the host bone, and the articular surface of the second metatarsal was shown radiographically to be well remodeled.

## Conclusion

Further studies with long-term follow-up are required to properly assess the usefulness of autologous osteochondral plug transplantation as a surgical arthroplastic option for preserving the MTP joint in patients with Freiberg's disease.

## Consent

Written informed consent was obtained from the patient for publication of this case report and any accompanying images. A copy of the written consent is available for review by the Editor-in-Chief of this journal.

## Competing interests

The authors declare that they have no competing interests.

## Authors' contributions

IN and HF examined the case file, reviewed the literature on Freiberg's disease, and made major contributions to the writing of the manuscript. TK and MK also contributed to the writing of the manuscript and its preparation for publication. All authors read and approved the final manuscript.
